# Gallbladder Volvulus Mimicking Immune Checkpoint Inhibitor-Related Acalculous Cholecystitis: A Case Report

**DOI:** 10.7759/cureus.116637

**Published:** 2026-09-21

**Authors:** Moussa Sylla, Samuel Bamogo, Eric Pappalardo

**Affiliations:** 1 Surgery, Centre Hospitalier Victor-Jousselin, Dreux, FRA

**Keywords:** acute acalculous cholecystitis, acute cholecystitis, gallbladder torsion, gallbladder volvulus, immune checkpoint inhibitors, laparoscopic cholecystectomy, pembrolizumab side effect, whirl sign

## Abstract

Gallbladder volvulus (GV) is a rare surgical emergency whose clinical and radiological presentation closely mimics acute cholecystitis. This diagnostic difficulty is amplified in patients receiving immune checkpoint inhibitors (ICIs), as gallbladder inflammation may initially be misattributed to an immune-related adverse event (irAE).

We report the case of an 82-year-old woman with microsatellite instability-high (MSI-H) duodenal adenocarcinoma treated with pembrolizumab. Following a temporary discontinuation of immunotherapy and initiation of corticosteroids for a suspected gastrointestinal irAE, she presented with acute right upper quadrant pain. Computed tomography (CT) showed a markedly distended, displaced gallbladder with absent mural enhancement. Initially suspected to be ICI-related cholecystitis, emergency laparoscopy instead revealed a >180° GV with transmural ischemic necrosis. A retrospective CT review identified a subtle cystic-pedicle "whirl sign." Following laparoscopic cholecystectomy, the patient recovered uneventfully, and pembrolizumab was later resumed.

GV should remain in the differential diagnosis of acute acalculous cholecystitis in elderly patients, even those on immunotherapy. A plausible immune-mediated explanation should not lead to diagnostic anchoring when clinical and radiological signs - such as ischemia or a whirl sign - indicate an acute mechanical surgical emergency.

## Introduction

Gallbladder volvulus (GV) is a rare but potentially life-threatening surgical emergency caused by torsion of the gallbladder around its vascular and biliary pedicle. Torsion initially compromises venous drainage and may subsequently impair arterial inflow, rapidly resulting in gallbladder ischemia, gangrene, and, if treatment is delayed, perforation [1-3]. GV predominantly affects elderly women and is usually associated with a mobile or “floating” gallbladder resulting from an elongated mesocyst or inadequate fixation of the gallbladder to the hepatic bed [3,4]. Although more than 500 cases have been reported, preoperative diagnosis remains challenging, and the diagnosis is frequently established only during surgical exploration [3,4].

The clinical presentation of GV often resembles acute cholecystitis, making early recognition difficult. Computed tomography (CT) may nevertheless provide important diagnostic clues, including marked gallbladder distension, an ectopic or displaced gallbladder, abnormal or absent mural enhancement suggestive of ischemia, and abnormalities of the gallbladder neck or cystic pedicle [1,5,6]. In particular, the “whirl sign” reflects twisting of the vascular and biliary structures of the cystic pedicle and may suggest gallbladder torsion. However, this finding can be subtle and may be overlooked when GV is not initially considered in the differential diagnosis [1,5,6].

This diagnostic challenge may be amplified in patients receiving immune checkpoint inhibitors (ICIs). Although uncommon, ICI-related cholecystitis has been reported and may present as an acalculous inflammatory process clinically and radiologically resembling conventional acute cholecystitis [7]. Therefore, when a patient receiving pembrolizumab develops right upper quadrant pain and gallbladder abnormalities, an immune-related adverse event (irAE) may represent a plausible initial diagnosis. Conversely, attributing new abdominal findings to immunotherapy may create a risk of diagnostic anchoring and premature closure, potentially obscuring an unrelated surgical emergency.

We report the case of an 82-year-old woman treated with immunotherapy for a microsatellite instability-high (MSI-H) duodenal adenocarcinoma who was admitted with a clinical picture of acute acalculous cholecystitis. The diagnosis of GV was established only during laparoscopic exploration. Retrospective review of the preoperative CT scan subsequently enabled identification of a subtle whirl sign of the cystic pedicle that had initially been overlooked. This observation highlights the diagnostic difficulties of this rare condition, the value of careful CT assessment, and the importance of considering GV in patients receiving immunotherapy who present with acute acalculous cholecystitis.

## Case presentation

An 82-year-old woman was admitted to the Emergency Department with acute abdominal pain that had progressively worsened over 48 hours. The pain had a sudden onset and was predominantly localized to the right upper quadrant. It was associated with nausea without vomiting. She reported no fever, jaundice, or change in bowel habits.

Her medical history included hypertension, hypothyroidism treated with levothyroxine, and a previous appendectomy. She was also being followed for MSI-H duodenal adenocarcinoma and had received pembrolizumab for three months. A week before the current admission, pembrolizumab had been temporarily discontinued because of gastric distension with suspected stenosis, and systemic corticosteroid therapy had been initiated at a dose of 40 mg daily. The digestive complication had raised concern for an irAE.

On admission, the patient was conscious, afebrile, and hemodynamically stable, with normal vital signs. Abdominal examination revealed diffuse tenderness, maximal in the right upper quadrant, without guarding, rigidity, or other signs of peritoneal irritation. There was no clinical jaundice.

Laboratory investigations revealed an inflammatory syndrome characterized by marked leukocytosis and a mildly elevated C-reactive protein. Liver function tests showed isolated mild hyperbilirubinemia, predominantly conjugated, whereas transaminases, alkaline phosphatase, and gamma-glutamyl transferase levels remained within normal limits. The comprehensive metabolic panel, including renal function and electrolytes, was unremarkable. The complete quantitative laboratory parameters and their respective reference ranges are summarized in Table 1.

**Table 1:** Comprehensive laboratory investigations at initial presentation

Parameter	Patient Value	Reference Range	Interpretation
White blood cell count	18,000 /mm³	4,000 - 10,000 /mm³	Increased
Hemoglobin	13.0 g/dL	12.0 - 16.0 g/dL	Normal
Hematocrit	39%	36 - 46%	Normal
C-reactive protein (CRP)	9 mg/L	< 5 mg/L	Increased
Sodium	137 mmol/L	135 - 145 mmol/L	Normal
Potassium	3.8 mmol/L	3.5 - 5.1 mmol/L	Normal
Bicarbonate	25 mmol/L	22 - 29 mmol/L	Normal
Calcium	2.21 mmol/L	2.10 - 2.60 mmol/L	Normal
Glucose (Random)	6.0 mmol/L	3.9 - 7.8 mmol/L	Normal
Creatinine	80 µmol/L	45 - 84 µmol/L	Normal
Total bilirubin	25 µmol/L	3 - 17 µmol/L	Increased
Conjugated bilirubin	15 µmol/L	< 5 µmol/L	Increased
Aspartate aminotransferase (AST)	24 U/L	10 - 40 U/L	Normal
Alanine aminotransferase (ALT)	26 U/L	10 - 40 U/L	Normal
Alkaline phosphatase (ALP)	45 U/L	40 - 120 U/L	Normal
Gamma-glutamyl transferase (GGT)	33 U/L	9 - 48 U/L	Normal

Emergency contrast-enhanced abdominal CT demonstrated a markedly distended gallbladder displaced from its hepatic bed. The gallbladder wall was thickened and showed absent contrast enhancement, consistent with parietal ischemia (Figure 1). No gallstones were identified. A moderate amount of intraperitoneal fluid was also present, without pneumoperitoneum or evidence of bowel ischemia or perforation.

**Figure 1:**
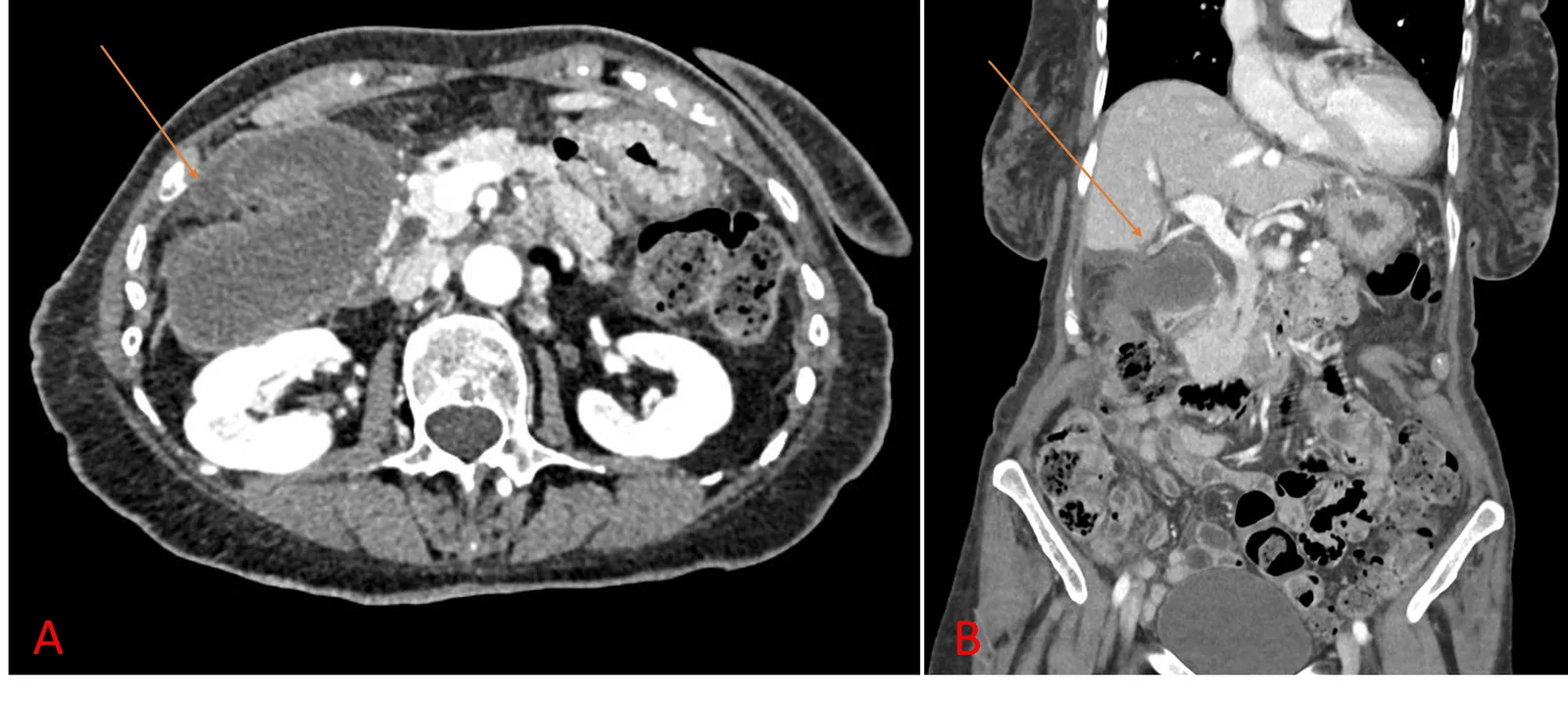
CT images A: axial slice; B: coronal slice: whirl sign of the cystic pedicle corresponding to twisting of the vascular and biliary structures (arrows).

Given the patient’s recent exposure to pembrolizumab, previous suspected immune-related digestive toxicity, and the radiological appearance of an ischemic acalculous gallbladder, acute acalculous cholecystitis potentially related to immunotherapy was initially considered. GV was not identified on the initial CT interpretation. Because of the suspected complicated acute cholecystitis and evidence of gallbladder ischemia, emergency laparoscopic exploration was performed.

Laparoscopy revealed an ischemic, distended gallbladder that was displaced from its normal anatomical position and attached to the liver by a complete mesocyst (Figure 2). The gallbladder was rotated more than 180° clockwise around the cystic pedicle, resulting in complete interruption of its blood supply and transmural ischemic necrosis. Approximately 400 mL of serosanguineous peritoneal fluid was aspirated. Systematic exploration of the abdominal cavity revealed no liver metastases, peritoneal carcinomatosis, or other abdominal abnormalities.

**Figure 2:**
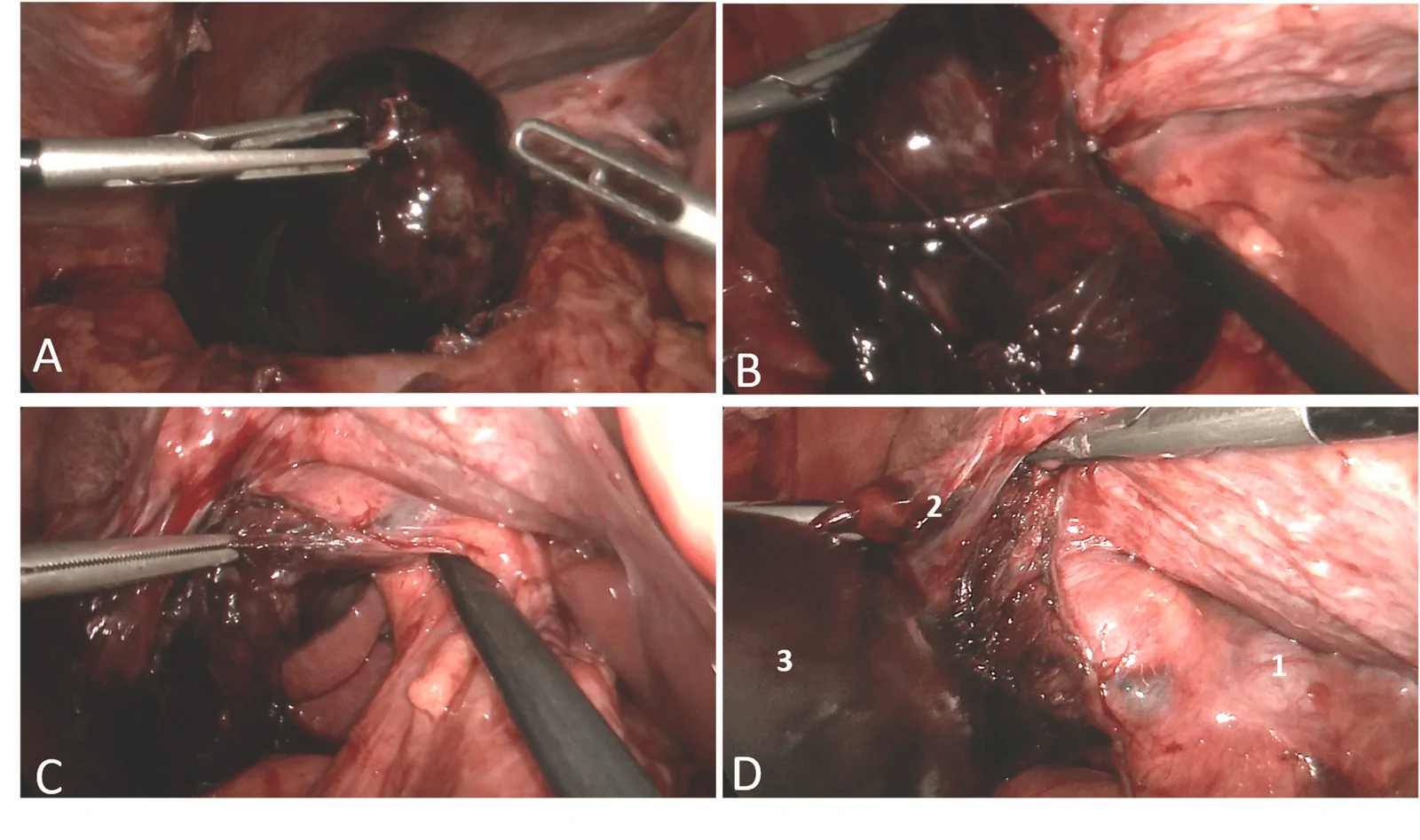
Intraoperative laparoscopic imaging A: Initial exploration showing an ischemic gallbladder. B: Beginning of counterclockwise gallbladder detorsion. C: End of detorsion showing a gallbladder with a long mesocyst. D: Final appearance after detorsion and beginning of dissection of Calot's triangle (1 = common bile duct, 2 = mesocyst, 3 = gallbladder).

Careful counterclockwise detorsion was first performed to restore the normal anatomy and allow clear identification of the cystic pedicle. Antegrade laparoscopic cholecystectomy was then completed according to standard surgical principles without technical difficulty. The peritoneal cavity was thoroughly irrigated, and two drains were placed, one in the subhepatic space and the other in the pouch of Douglas.

Histopathological examination demonstrated ischemic cholecystitis with transmural hemorrhagic infarction. No dysplastic lesion or tumor infiltration was identified.

Following the intraoperative diagnosis, the preoperative CT scan was retrospectively reviewed. A subtle whirl sign involving the cystic pedicle, corresponding to twisting of the vascular and biliary structures, was identified (Figure 1). This finding had not been recognized during the initial radiological interpretation.

The postoperative course was uneventful. Bowel function, oral intake, and mobilization were resumed early. The drains were removed because of their low output, and the patient was discharged on postoperative day 4. At more than three months of follow-up, she remained asymptomatic without postoperative complications or recurrence. Pembrolizumab was resumed three months after surgery without a reported biliary or other specific adverse event.

## Discussion

GV is an uncommon cause of acute abdomen characterized by torsion of the gallbladder around its vascular and biliary pedicle. The resulting vascular compromise may progress rapidly from venous congestion to arterial ischemia, transmural necrosis, gangrene, and perforation [8-10]. The anatomical substrate is usually a mobile gallbladder with insufficient attachment to the hepatic bed, most commonly related to an elongated mesocyst. In elderly patients, hepatic atrophy, loss of visceral fat, and decreased tissue elasticity may further increase gallbladder mobility and facilitate torsion [3,8,11].

Our patient exhibited several features consistent with this pathophysiological mechanism. She was an elderly woman, and laparoscopic exploration demonstrated a floating gallbladder with a complete mesocyst and a clockwise rotation of more than 180° around the cystic pedicle. Complete interruption of the blood supply resulted in transmural ischemic necrosis. The absence of gallstones was also compatible with GV, which may occur without cholelithiasis [4,12,13]. Thus, in an elderly patient presenting with acute acalculous gallbladder inflammation, particularly when imaging suggests ischemia, GV should remain in the differential diagnosis even in the absence of gallstones.

The main diagnostic challenge is the substantial clinical overlap between GV and acute cholecystitis. Patients may present with acute right upper quadrant pain, nausea, vomiting, leukocytosis, and variable inflammatory abnormalities. Consequently, despite the increasing use of cross-sectional imaging, preoperative diagnosis remains inconsistent, and the condition is still frequently diagnosed intraoperatively [4,8,14]. In our patient, CT demonstrated a markedly distended gallbladder displaced from its hepatic bed, with wall thickening and absent mural enhancement. These findings indicated severe gallbladder ischemia but were initially interpreted in the context of acute acalculous cholecystitis rather than mechanical torsion.

CT may nevertheless provide important clues when the position, orientation, and vascular pedicle of the gallbladder are specifically assessed. Reported findings include a displaced or ectopic gallbladder, marked distension, abnormal or absent mural enhancement, a beak configuration of the gallbladder neck, and twisting of the cystic pedicle producing a whirl sign [1,5,6]. Recent literature has further emphasized the diagnostic value of recognizing characteristic combinations of these findings [1]. In our patient, a subtle whirl sign involving the cystic pedicle was present on the initial CT but was not recognized prospectively. It became apparent only after the intraoperative diagnosis of GV prompted retrospective review. This illustrates that even when a characteristic radiological sign is present, the diagnosis may be missed if torsion is not included in the initial differential diagnosis.

The difficulty of preoperative diagnosis remains evident in recent literature. In a systematic review of 44 cases published between 2020 and 2024, Salati et al. reported a preoperative diagnosis in only 41% of cases despite the increasing availability of modern imaging techniques [14]. Recent case reports and reviews have similarly emphasized that GV may remain unrecognized until surgery [8,11,15,16]. The practical implication is that CT assessment in an elderly patient with suspected acute acalculous cholecystitis should extend beyond the presence of gallbladder wall inflammation. Gallbladder position, orientation, mural perfusion, and the morphology of the cystic pedicle should also be evaluated. The coexistence of gallbladder displacement and absent mural enhancement, particularly when accompanied by a whirl sign, should raise suspicion for a mechanical vascular cause of gallbladder ischemia.

Once GV is suspected, definitive treatment should not be delayed because progression to gangrene and perforation may occur rapidly. Cholecystectomy is the definitive treatment, and laparoscopic surgery is increasingly used when feasible [5,17]. In our patient, emergency surgical exploration was undertaken because of the suspected complicated acute acalculous cholecystitis and, importantly, the CT evidence of gallbladder ischemia. Although the volvulus itself was not diagnosed preoperatively, urgent surgery allowed definitive treatment before perforation or generalized biliary peritonitis occurred.

The distorted anatomy associated with torsion requires particular attention during surgery. Detorsion before definitive dissection may restore the normal orientation of the gallbladder and cystic pedicle and facilitate identification of the cystic duct and vascular structures [10,17]. In our patient, careful detorsion was performed before dissection, followed by antegrade laparoscopic cholecystectomy without technical difficulty despite extensive transmural ischemic necrosis. Histopathological examination demonstrated ischemic cholecystitis with transmural hemorrhagic infarction and no dysplastic lesion or tumor infiltration, confirming that the gallbladder abnormality resulted from vascular compromise rather than malignant involvement.

The immunotherapeutic context represents the distinctive diagnostic challenge of this case. The patient had MSI-H duodenal adenocarcinoma and was receiving pembrolizumab. Before the acute biliary presentation, pembrolizumab had been temporarily discontinued because of gastric distension with suspected stenosis, and systemic corticosteroid therapy had been initiated at a dose of 40 mg daily. Consequently, when a new gallbladder abnormality developed, an immune-mediated adverse event represented a clinically plausible explanation. This possibility is supported by the recognized occurrence of ICI-associated cholecystitis. Abu-Sbeih et al. reported 25 cases of cholecystitis considered related to ICI therapy among 4,253 patients receiving ICIs, corresponding to an incidence of approximately 0.6% [7]. ICI-associated cholecystitis may present with clinical and radiological findings resembling conventional acute cholecystitis, making differentiation from other causes of gallbladder inflammation challenging.

However, this context may also create a diagnostic pitfall. When a patient receiving an ICI develops a new inflammatory abnormality, there is a risk of attributing the findings to treatment toxicity before fully excluding an unrelated surgical condition. In the present case, the recent interruption of pembrolizumab and the use of corticosteroids for a suspected gastrointestinal complication made an immune-mediated explanation particularly plausible. Nevertheless, the marked displacement of the gallbladder and absent mural enhancement were signs of severe ischemia that warranted urgent consideration of a mechanical vascular cause. The eventual discovery of complete gallbladder torsion demonstrated that the underlying pathology was mechanical rather than immune-mediated.

Importantly, GV has previously been reported in a patient receiving immunotherapy. Wuheb et al. described GV in an elderly patient receiving immunotherapy, with CT demonstrating an abnormally positioned distended gallbladder and a swirl sign involving the cystic duct and artery pedicle [18]. Therefore, our case should not be considered the first reported association between GV and immunotherapy. Its clinical value instead lies in the particular diagnostic circumstances in which GV mimicked a possible ICI-related acalculous cholecystitis. In our patient, this diagnostic challenge was reinforced by the fact that pembrolizumab had already been interrupted and corticosteroids initiated for a suspected gastrointestinal treatment-related complication. The subsequent gallbladder ischemia could consequently be interpreted within an established framework of immune toxicity, whereas the actual mechanism was acute mechanical torsion.

Another distinctive feature of our case is the retrospective recognition of the cystic pedicle whirl sign. Unlike cases in which the whirl sign immediately raises suspicion for GV, the sign in our patient was subtle and initially overlooked. Its recognition after surgery provides an important radiological and cognitive lesson: interpretation of imaging is influenced not only by the visibility of a sign but also by the diagnostic hypotheses considered at the time of interpretation. In patients receiving immunotherapy, maintaining a broad differential diagnosis remains essential when imaging demonstrates gallbladder displacement or ischemia.

Overall, this case emphasizes that GV should remain in the differential diagnosis of acute acalculous cholecystitis in elderly patients, including those receiving ICIs. In this setting, the presence of a displaced gallbladder, reduced or absent mural enhancement, and abnormalities of the cystic pedicle should prompt careful evaluation for torsion. Although the diagnosis may ultimately require surgical exploration, recognition of characteristic CT findings, particularly the whirl sign, may facilitate earlier suspicion and timely intervention. More broadly, a plausible immune-mediated explanation should not lead to premature diagnostic closure when clinical and radiological findings remain compatible with an acute mechanical surgical emergency.

## Conclusions

GV is a rare surgical emergency that should be considered in elderly patients presenting with acute acalculous cholecystitis, particularly when imaging demonstrates gallbladder displacement or ischemia. In patients receiving ICIs, the diagnostic challenge may be greater because gallbladder inflammation can initially be attributed to an irAE. Careful assessment of the gallbladder position, mural enhancement, and cystic pedicle, including the search for a whirl sign, may raise suspicion for torsion. When gallbladder ischemia is suspected, early surgical exploration and cholecystectomy remain essential to prevent progression to gangrene or perforation. This case emphasizes that a plausible immune-mediated explanation should not lead to premature diagnostic closure when clinical and radiological findings remain compatible with an acute mechanical surgical emergency.
